# Lunasin: A Novel Cancer Preventive Seed Peptide

**DOI:** 10.4137/pmc.s372

**Published:** 2008-03-25

**Authors:** Blanca Hernández-Ledesma, Ben O. de Lumen

**Affiliations:** Department of Nutritional Sciences and Toxicology, University of California Berkeley, CA, 94720-3104, U.S.A

**Keywords:** lunasin, soy protein, seeds, cancer prevention

## Abstract

Cancer is one of the leading causes of deaths in the Western world. Approximately one-third of these deaths are preventable by lifestyle factors, including modification of nutritional habits. Studies have demonstrated that adequate nutrition with certain types of foods containing bioactive compounds might offer significant protection against carcinogenesis. Soybeans contain a variety of phytochemicals with demonstrated anticancer activity, including isoflavones, protease inhibitors, and more recently lunasin, a novel cancer preventive seed peptide. Initially isolated from soybean, lunasin has also been reported in barley and wheat. The purpose of this review is to summarize the most recent evidence on the possible benefits of lunasin for cancer prevention.

## Introduction

Soybean (*Glycine max*) is a legume consumed worldwide, but most commonly in Asian countries where it is a staple used to prepare both fermented and non-fermented foods. Asians consume an average of 20 to 80 g of traditional soy foods daily, the most common of which are tofu, miso and tempeh. Recently, soybean foods have generated a lot of interest because of reported beneficial effects on nutrition and health. It has been demonstrated that Asian populations consuming large amounts of soybean products have a lower risk of osteoporosis and some chronic diseases, most notably heart disease and cancer (McCue and Shetty, 2004). Epidemiological studies, animal experiments and *in vitro* studies have shown that soy products are associated with decreased risk for prostate (Jacobsen et al. 1998; Lee et al. 2003), breast (Wu et al. 1998; Dai et al. 2002; Yamamoto et al. 2003) and endometrial cancers (Goodman et al. 1997).

Soybean contains a variety of phytochemicals with demonstrated anticancer activity, including protease inhibitors, inositol hexaphosphate (phytic acid), β-sitosterol, saponins, and isoflavones (Messina and Barnes, 1991). The most widely studied bioactive substances in soy are Bowman-Birk protease inhibitor (BBI) and the isoflavones. BBI is a serine protease inhibitor with a well-characterized ability to inhibit trypsin and chymotrypsin. It has been shown to have anticarcinogenic effects on many different cell lines (Yavelow et al. 1985; Billings and Habres, 1992; Kennedy, 1998a, b; Zhang et al. 1999; Meyskens, 2001; Kennedy and Wan, 2002). Its capacity for preventing or suppressing carcinogenic processes has been also demonstrated in a wide variety of *in vitro* and *in vi*v*o* animal model systems. In preclinical studies, BBI has been found to interfere effectively with the development of tumors induced by chemical carcinogens in the lung or gastrointestinal tract of mice (Witschi and Kennedy, 1989; St Clair et al. 1990; Kennedy et al. 1996), the esophagus and colon of rats (von Hofe et al. 1991; Kennedy et al. 2002) and the oral cavity of hamsters (Messadi et al. 1986) and with radiation-induced lymphosarcoma in mice (Evans et al. 1992). As a result of this evidence, BBI acquired the status of an “investigational new drug” from the FDA in 1992 and currently is being evaluated in large-scale human trials as an anticarcinogenic agent in the form of BBI concentrate (BBIC). The results of phase I and II clinical trials have shown that BBIC has a substantial positive clinical effect in patients with oral leukoplakia (Armstrong et al. 2000, 2003; Meyskens, 2001). At this time, a Phase IIb randomized, placebo-controlled clinical trial to determine the clinical effectiveness of BBIC is under way. BBI and BBIC evidently works by inhibiting proteases involved in initiation and promotion of carcinogenesis (Kennedy, 1994), but the biochemical and molecular bases for this mechanism of action need to be further elucidated.

The chemopreventive properties of soybean isoflavones, which are phytoestrogens, have been attributed to different biological activities, mainly to their long-term estrogenic effects and their antioxidant activity (McCue and Shetty, 2004). The administration of soy isoflavones in a soy protein matrix has raised the possibility that other proteins contribute to the observed preventive effects attributed to isoflavones mixtures (Pollard and Wolter, 2000). These observations gave special importance to the discovery of the cancer preventive properties of the peptide lunasin discovered in our laboratory.

## Discovery of Lunasin

Lunasin is a unique 43-amino acid peptide which sequence is the following: S K W Q H Q Q D S C R K Q K Q G V N LT P C -EKHIMEKIQG-*RGD*-**DDDDDDDD**

This peptide contains 8 Asp (D) residues in its carboxyl end (bold) preceded by a cell adhesion motif Arg-Gly-Asp (RGD) (italics) and a predicted helix (underlined) with structural homology to a conserved region of chromatin-binding proteins.

Lunasin was initially identified in the soybean cotyledon when a cDNA encoding a post-translationally processed 2S albumin (Gm2S-1) was cloned from mid-maturation soybean seed (Galvez et al. 1997). Gm2S-1 codes for a signal peptide, a small subunit (called lunasin, from the Tagalog word “lunas” for cure), a linker peptide, and a large subunit methionine-rich protein. Transfection and expression of the lunasin gene inside mammalian cells result in mitotic arrests leading to cell death (Galvez and de Lumen, 1999). This antimitotic effect of this peptide has been attributed to the binding of its poly-D carboxyl end to regions of hypoacetylated chromatin, such as that found in kinetochores in centromeres. As a result, the kinetochore complex does not form properly, and the microtubules fail to attach to the centromeres, leading to mitotic arrest and eventually to cell death (Galvez and de Lumen, 1999).

## Lunasin in Soybean and Other Seeds

A screening of the U.S. Department of Agriculture soybean germplasm collection showed that lunasin is present in the 144 analyzed soy genotypes in concentrations ranging from 6 to 7 mg of lunasin/g of protein (Gonzalez de Mejia et al. 2004). Jeong and co-workers have analyzed different Korean varieties of soybean which contain lunasin concentrations ranging from 4.40 to 70.49 mg of lunasin/g of protein (Jeong et al. 2007a). These variations indicate that the levels of this peptide in soybeans can be genetically manipulated and suggest the possibility of selecting and breeding varieties of soy with higher lunasin content. It has been also demonstrated that large-scale processing of soy to produce different soy protein fractions influences lunasin concentration. This concentration varies from 12 to 44 mg lunasin/g of flour in different U.S. commercially available soy proteins (Gonzalez de Mejia et al. 2004). The stages of seed development and sprouting also affect the concentration of lunasin in the soybean (Park et al. 2005). A notable increase of lunasin content occurs during seed maturation. However, sprouting leads to a continuing decrease of lunasin with soaking time. Light and dark conditions do not seem to affect the content of this peptide (Park et al. 2005). These data are useful in the preparation of soy fractions enriched in lunasin as well as in recommendations of dietary intakes of lunasin.

In search of natural sources of lunasin besides soybean, the identification, isolation and bioassay of lunasin from barley (Jeong et al. 2002) and wheat (Jeong et al. 2007b) have been reported. The amount of lunasin in the crude extracts of eight varieties of wheat ranges from 211 to 249 μg lunasin/g of seed. Such varying concentrations suggest the availability of lunasin in wheat food products to consumers (Jeong et al. 2002). Recently, lunasin has been also found in the *Solanaceae* family (Jeong et al. 2007c), amaranth (Silva-Sanchez et al. 2008), and pepper (unpublished data).

Lunasin is a very heat stable peptide, surviving and retaining its activity even after 10 min of boiling (de Lumen, 2005). *In vitro* digestibility studies have demonstrated that pure synthetic lunasin is digested by pancreatin (Galvez et al. 2001). However, animal studies using ^3^H-labeled synthetic lunasin have shown that about the 35% of the oral dose is absorbed and ends up in the various tissues of mice and rats 6 hours after administration by gavage together with lunasin-enriched soy protein (de Lumen, 2005). Jeong and co-workers have studied *in vivo* digestibility of a lunasin-enriched soy (LES) in rats. These rats were fed LES for 4 weeks and the liver and blood were analyzed for lunasin that was extracted in an intact and bioactive form (Jeong et al. 2007a). Similar results were found when rats were fed lunasin-enriched wheat (Jeong et al. 2007b). These observations suggest that in soy and wheat, naturally occurring protease inhibitors, such as BBIC and Kunitz Trypsin Inhibitor (KTI) protect lunasin from digestion in the gastrointestinal tract of humans and animals. The capacity of a compound to be absorbed after being orally administered and to reach the target tissues in a bioactive state is one of the most important features of an ideal cancer preventive agent.

## In Vitro and in Vivo Effects of Lunasin

Although its physiological significance remains to be established, lunasin appears to be an ideal chemopreventive agent. Its chemopreventive properties both *in vitro* and *in vivo* have been demonstrated. In the absence of carcinogens, lunasin peptide added exogenously does not seem to affect cell morphology and proliferation but prevents transformation in the presence of carcinogens. At nanomolar concentrations, lunasin added exogenously to mouse fibroblasts cells C3H10T1/2 significantly suppresses foci formation induced by the chemical carcinogens DMBA and MCA (Galvez et al. 2001). Colony formation is a measure of anchorage-independent cell growth, one of the characteristics of transformed cells. Lunasin suppresses colony formation in NIH3T3 cells, where it is 4-fold more effective, on a molar basis, than the BBI, a known cancer-preventive agent from soy (Park et al. 2005).

Lunasin also prevents transformation of mammalian cells by viral oncogenes. It inhibits, in a dose-dependent manner, foci formation in C3H cells and NIH3T3 cells transfected with the oncogene E1A, known to induce cell proliferation by inactivating the tumor suppressor protein Rb (Lam et al. 2003). Interestingly, lunasin is effective even when added up to 15 days after transfection with E1A gene, suggesting its efficacy when applied even after the transformation event. Lunasin also suppresses colony formation induced by the *ras*-oncogene in MCF-7 cells stably transfected with an inducible form of the oncogene (Jeong et al. 2003).

The effect of Lunasin on cancer prevention has been also demonstrated using an *in vivo* mouse model. In this model, dermal application of lunasin at 250 μg/week reduced skin tumor incidence in SENCAR mice treated with DMBA and TPA by approximately 70% compared with the untreated control (Galvez et al. 2001). This treatment also reduced the tumor multiplicity (tumor/mouse), and delayed by two weeks the appearance of papilloma in the mice relative to the untreated control. Using a 2H_2_O-labeling method to measure cell proliferation *in vivo*, it has been found that lunasin slows down epidermal cell proliferation in mouse skin in the absence and presence of DMBA (Hsieh et al. 2004).

## Epigenetic Mechanism of Action

**Figure 1:**
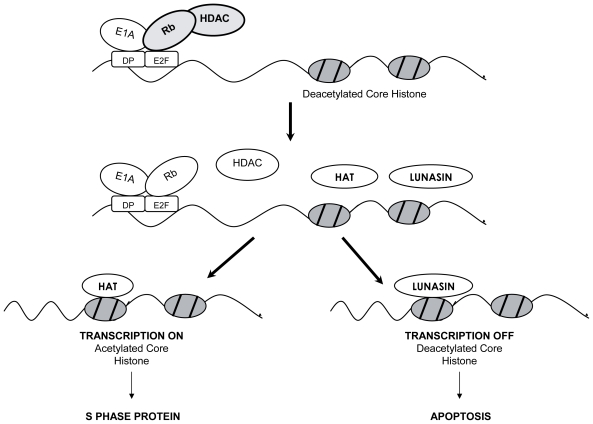
E1A-Rb-HDAC model to explain the ability of Lunasin to suppress E1A-induced transformation. Top diagram: Rb controls G1/S transcription by interacting with E2F promoter and recruiting HDAC to keep the core histones in the deacetylated (repressed) state. In a cell being transformed, E1A inactivates Rb and dissociates Rb-HDAC complex, exposing the deacetylated core histones in the E2F promoter (middle diagram). Lunasin competes with histone acetyltransferase (HAT) in binding to the deacetylated core histones. Bottom diagram: HAT binds and acetylates core histones, turning on E2F cell cycle transcription factors, leads to cell proliferation. Lunasin binds, turns off the transcription, perceived as abnormal by cell, results in apoptosis.

Chemical carcinogenesis and viral oncogenesis share common mechanism(s) involving changes in chromatin status that lunasin disrupts to suppress cancer formation. Lunasin peptide added exogenously to mammalian cells treated with the histone deacetylases inhibitor sodium butyrate, internalizes into the cell and crosses the nuclear membrane, localizing in the nucleus. There, it binds specifically to deacetylated core histones H3 and H4, inhibiting their acetylation (Galvez et al. 2001; Jeong et al. 2002). Recently, the histone acetylation inhibitory properties of different soybean and wheat varieties have been demonstrated (Jeong et al. 2007a, b). As expected, the degree of inhibition correlated with lunasin concentration. Once extracted and purified, lunasin shows properties of inhibiting core histone acetylation. It is effective at 10 nM, bringing about a 20% and 25% reduction in acetylated histones H3 and H4, respectively. At 1000 nM, the acetylation reduction is 80% for H3 histone and 75% for H4 histone, relative to the control (Jeong et al. 2007a). The affinity of lunasin for hypoacetylated chromatin and its inhibitory effect on histone acetylation is relevant to the proposed epigenetic mechanism of action using the E1A-Rb-HDAC model (Fig. 1, de Lumen, 2005). This model stipulates that lunasin selectively kills cells that are being transformed by disrupting the dynamics of histone acetylation-deacetylation when a transforming event occurs. The tumor suppressor protein Rb functions by interacting with E2F promoter and recruiting histone deacetylase (HDAC) to keep the core histones in the deacetylated (repressed) state. The inactivation of Rb by the oncoprotein E1A dissociates the Rb-HDAC complex, exposing the deacetylated core histones for acetylation by histone acetyltransferases (HATs). When this event occurs, lunasin is triggered into action and binds to the deacetylated core histones competing with the HATs and turning off transcription. We propose that the binding of lunasin to deacetylated core histones disrupts the dynamics of histone acetylation-deacetylation, which is perceived as abnormal by the cell and leads to apoptosis. This epigenetic mechanism suggests that lunasin can influence regulatory pathways involving chromatin modifications that may be fundamental to carcinogenic pathways in general, suggesting that lunasin could be effective against a number of cancers that involve chromatin modification. Interestingly, the tumor suppressors Rb, p53 and pp32, function partly through chromatin modification. We propose that when these tumor suppressors are inactivated lunasin takes over as a surrogate tumor suppressor and selectively kills cells that are being transformed.

The mechanism by which lunasin inhibits histone acetylation is not definitively known. Evidently, lunasin binds to deacetylated histone by ionic interaction with its negatively charged poly-D. Deletion of the coding region for poly-D in the lunasin cDNA nullifies its antimitotic activity when transfected into mammalian cells (Galvez and de Lumen, 1999). The N-terminus of lunasin that includes the helical region may play a role in targeting lunasin to deacetylated histones (Galvez et al. 2001). Experiments are being carried out to elucidate the histone acetylation inhibitory mechanisms of lunasin.

## Future Perspectives

Lunasin is a novel and promising chemopreventive peptide derived from soybean, wheat, barley, and other plant seeds. Its demonstrated cancer preventive properties *in vitro* make lunasin a perfect candidate to exert an *in vivo* cancer-preventive activity. Further research is needed to demonstrate this activity. The preventive efficacy of lunasin administered in the diet and other routes needs be tested against different types of cancer. In our laboratory, animal studies demonstrating this efficacy against colon, prostate and breast cancer are currently being carried out. Moreover, the proposed epigenetic mechanism of action suggesting that lunasin can be effective against cancers where chromatin modification is involved in carcinogenesis needs to be further elucidated through genomics and proteomics. There is still much to be learned about the effects of lunasin on cancer risks and this area of research holds considerable potential.
